# Study on the Red Blood Cell Distribution Width in Connective Tissue Disease Associated with Interstitial Lung Disease

**DOI:** 10.1155/2020/8130213

**Published:** 2020-01-24

**Authors:** Chuanmei Liu, Jie Yang, Zhiwei Lu

**Affiliations:** Department of Respiratory Medicine, Yi Ji Shan Hospital of Wannan Medical College, Wuhu, Anhui, China

## Abstract

**Background:**

Connective tissue disease (CTD) associated with interstitial lung disease (ILD) affects the lungs and can lead to considerable morbidity and shortened survival. Red blood cell distribution width (RDW) is a readily available parameter that is routinely reported with complete blood cell count (CBC) This study aimed to investigate the predictive value of RDW in CTD-ILD.

**Methods:**

A retrospective analysis was performed on 180 patients with CTD-ILD and 202 patients with CTD but without ILD between April 2016 and December 2018. Baseline demographics, laboratory results, imaging examinations, and results of ultrasound scans were analysed.

**Results:**

In comparison with patients without ILD, patients with CTD-ILD displayed a larger RDW (14.65 ± 2.08 vs. 14.17 ± 1.63, *P*=0.002), and RDW shared positive relationships with pulmonary artery systolic pressure (*r* = 0.349; *P*=0.002), and RDW shared positive relationships with pulmonary artery systolic pressure (*r* = 0.349; *P*=0.002), and RDW shared positive relationships with pulmonary artery systolic pressure (*r* = 0.349; *P*=0.002), and RDW shared positive relationships with pulmonary artery systolic pressure (*P*=0.002), and RDW shared positive relationships with pulmonary artery systolic pressure (*P*=0.002), and RDW shared positive relationships with pulmonary artery systolic pressure (*P*=0.002), and RDW shared positive relationships with pulmonary artery systolic pressure (

**Conclusions:**

RDW was significantly increased in patients with CTD-ILD under various CTD backgrounds and may be a promising biomarker that may help physicians predict CTD-ILD risk.

## 1. Introduction

Connective tissue disease (CTD) is characterised by autoimmune-mediated damage that involves various systems, such as skin, joints, muscles, heart, kidneys, and lung. CTDs include rheumatoid arthritis (RA), systemic sclerosis (SSc), dermatomyositis/polymyositis (PM/DM), Sjogren's syndrome (SS), systemic lupus erythematosus (SLE), and undifferentiated and mixed connective tissue disease. Interstitial lung disease (ILD) commonly occurs in the lung parenchyma, the airways, pulmonary vasculature, and structures of the chest wall. CTD associated with ILD (CTD-ILD) is attributed to patients who met the diagnosis criteria for ILD and experience the recurrence of CTD simultaneously [1, 2]. CTD-ILD is a common and serious form of secondary ILD and can lead to significant morbidity and shortened survival. Pulmonologists should evaluate evidence of underlying CTD in all patients with ILD.

Red blood cell distribution width (RDW) is a parameter that reflects the heterogeneity of circulating erythrocyte volume and is used for the differential diagnosis of anemia. RDW has been associated with various diseases in previous studies, and it is a promising biomarker for cardiovascular disease [3, 4], community-acquired pneumonia (CAP) [5], chronic obstructive pulmonary disease (COPD), and other acute or chronic conditions [6, 7]. RDW is a predictor of survival in idiopathic pulmonary fibrosis (IPF) [8].

Several studies have examined the relationship between RDW and CTD or ILD [8–10]. However, information about the relationship between the RDW and CTD-ILD in CTD-ILDs is limited despite the availability of various works on CTD. Moreover, the association between RDW and the clinical parameters of CTD-ILD has not been established. In this study, we analysed RDW's relationship with multiple clinical factors in CTD-ILD patients.

## 2. Methods

### 2.1. Patients

In this study, follow-ups were carried out among 382 patients diagnosed with CTD at the Yi Ji Shan Hospital of Wannan Medical College from April 2016 to December 2018. Each CTD patient underwent high-resolution CT (HRCT) scan of the lungs for the evaluation of ILD. Among them, 180 patients, who received a diagnosis of CTD-ILD, were diagnosed according to published guidelines [11]. A total of 202 CTD patients were used as the control group to maximise the elimination of distractions. CTD diagnoses were carried out based on relevant international diagnostic guidelines [12–17]. Exclusion criteria were as follows: acute coronary syndromes, infection, malignancy, heart failure, severe anemia, malnutrition, blood transfusion, hematological disorders, iron deficiency anemia, iron supplementation therapy, thromboembolic disease, cerebrovascular disease, and severe liver or renal insufficiency.

### 2.2. Data Collection

Demographics characteristics, including age, sex, length of hospital confinement (days), and general medical history, were recorded. Laboratory parameters, including complete blood count, RDW (normal range: 11.5%–14.5%), hemoglobin (Hb), white blood cell (WBC), blood urea nitrogen (BUN), creatinine (Cr), C-reactive protein (CRP), echocardiography, and high-resolution CT findings, were obtained from patient's medical records, while pulmonary artery systolic pressure (PASP) was measured using the echocardiography method.

### 2.3. Statistical Analysis

The distribution of values was assessed using the Kolmogorov–Smirnov test. Continuous variables are presented as means ± SD using Student's *t* test. Categorical variables were expressed as percentage (%) or number (*n*) and were compared using the chi-square statistic. Pearson's correlation coefficient analysis was used to evaluate the correlations between RDW and the other laboratory variables. Logistic regression analysis was applied to determine associations between laboratory indicators and CTD-ILD risk. Variables that were significant (*P* < 0.05) in the univariate analysis were included in the multivariate logistic regression analysis and were used to determine which factors had the most effect on CTD-ILD. The receiver operating characteristic (ROC) curve analysis was used to assess the predictive value of RDW on sensitivity, specificity, and the area of AUC. *P* values less than 0.05 were considered statistically significant. All data calculations were performed using the statistical analysis software (version 19.0, SPSS, IBM Corporation).

## 3. Results

A total of 382 eligible CTD patients were divided into two groups according to lung conditions. The first group included 180 CTD patients with ILD, and the 202 cases without ILD comprised the control group. The baseline demographic and clinical characteristics of the patients are summarised in Table 1. The RDW was higher in patients with CTD-ILD (14.65 ± 2.08 vs. 14.17 ± 1.63, *P*=0.012) than in the controls. No significant differences were found in the WBC, ALB, SOD, length of hospital confinement, and smoking history between the CTD and CTD-ILD groups. However, the mean age of patients with CTD-ILD was higher than that of patients with only CTD (52.91 ± 11.58-year-old vs. 49.93 ± 13.76-year-old, *P*=0.024) with a slight male predominance (17.22% vs. 9.41%, *P*=0.024).

In the CTD-ILD group, the association between RDW and other parameters was assessed using correlation analysis, as shown in Table 2. Statistical analysis showed that RDW and Hb (*r* = −0.252, *P*=0.001), RDW and PLT (*r* = −0.183; *P*=0.014), RDW and ALB (*r* = −0.241; *P*=0.001), and RDW and SOD (*r* = −0.274; *P* < 0.001) were negatively correlated. In addition, the study found a positive correlation between RDW and PASP (*r* = 0.349; *P* < 0.001), RDW and length of hospital confinement (*r* = 0.172; *P*=0.022), and RDW and hospitalisation expenses (*r* = 0.158; *P*=0.037).

The results of logistic regression analysis are shown in Table 3. Univariate analysis indicated that RDW, Hb, IgG, C3, age, sex, hospitalisation expenses, and number of readmissions in 1 year differed significantly between patients with CTD and CTD-ILD (*P* < 0.05). Multivariate analysis included relevant variables with *P* values < 0.05 and clinical significance in univariate analysis. These indicators were then entered into the multivariate logistic regression analysis, which identified RDW (odds ratio (OR): 1.232, 95% confidence interval (CI): 1.053–1.422, *P*=0.009), IgG (OR: 1.103, 95% CI: 1.051–1.159, *P* < 0.001), and age (OR: 1.032, 95% CI: 1.010–1.054, *P*=0.004) as independent predictors of CTD-ILD risk.

**Figure 1:**
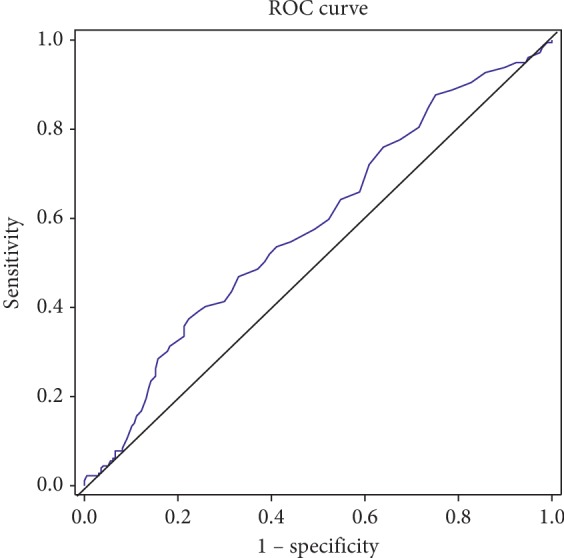
ROC analysis for RDW predicting CTD with ILD.

Receiver operating characteristic (ROC) curve analysis was used to determine the best RDW value for CTD-ILD, and the AUC of the RDW was 0.584 (95% CI: 0.526–0.641, *P*=0.005), as shown in Figure 1. The value of 14.85% was taken as the optimal RDW cut-off value in the CTD-ILD group with a sensitivity of 41.2% and a specificity of 75.2%.

## 4. Discussion

Our study was the first to report the relationship between RDW and other data among patients with CTD-ILD under various CTD backgrounds. For many years, RDW has been almost exclusively used for the differential diagnosis of anemia. Recently, many researchers are focusing on the use of RDW in different human disorders. RDW has been broadly investigated in cardiovascular disorders, CAP, and COPD. RDW has been used as a biomarker for predicting adverse outcomes in these conditions [3–6, 18]. However, the underlying mechanisms of RDW elevation in these diseases are not well known. RDW is associated with various CTD backgrounds, including SLE, RA, and SSc [9, 10]. However, RDW studies that considered ILD patients are limited. Nathan demonstrated that RDW could be a biomarker for IPF outcomes [8].

The importance of RDW has been recently recognised in patients with CTD-ILD. We demonstrated that the RDW value, which is a simple and inexpensive parameter, is significantly higher in patients with CTD-ILD than in those without ILD. The condition of CTD-ILD might reflect the chronic inflammatory state and the circulating levels of cytokines, such as tumour necrosis factor α, interleukin-1, and IL-6 [19]. In addition, the data revealed a positive association between the RDW and PASP and the RDW and length of hospital confinement in CTD-ILD. Half of cases with pulmonary arterial hypertension occurrence were associated with CTD [20]. A mean pulmonary arterial pressure (MPAP) between 21 and 24 mmHg is clinically relevant and affects patient outcomes in IPF [21, 22]. Patients undergo echocardiography to measure pulmonary systolic blood pressure instead of the invasive right heart catheterisation as an evaluation factor because of limited testing conditions and high risks and complications in the latter. Our study found a positive correlation between RDW and PASP in CTD-ILD. Moreover, significant differences were found between the two groups, in which patients with ILD had significantly higher PASP values (30.92 ± 11.12 vs. 26.80 ± 5.91, *P*=0.001). Echocardiography, which is a noninvasive and simple operation, can be used for the early screening and follow-up for CTD-ILD. We conducted follow-up for 1 year among patients and found that patients with CTD-ILD have higher readmission times than those without ILD, thereby indicating that the disease control in these patients is not good, and they need to be hospitalised repeatedly. This condition has increased the economic burden to our society. Our research results may have important clinical implications for the use of RDW value in predicting CTD-ILD and may provide more contributions to future studies.

Our study has several significant limitations. Firstly, it is a small retrospective review at a single center, and we showed the RDW was higher in the study group and it played an important role in the diagnosis of CTD-ILD, but most of patients without RDW level change data after treatment, which could not reflect the important role of RDW values in the treatment. Our study did not collect serum ferritin and serum iron from all the patients, so we did not know the iron status from all the patients directly. However, we collect the hematocrit (Hct), mean corpuscular volume (MCV), mean corpuscular hemoglobin (MCH), and mean corpuscular hemoglobin concentration (MCHC). These indicators exclude iron deficiency anemia, and it could reflect the iron status from a side face. Thus, the sample size is limited. Our results require confirmation through further studies using multicenter, prospective, and large-scale cohorts. Secondly, the age and sex of the two groups were not well matched. The age of patients in the CTD-ILD group was higher than that in the other groups, which might explain the high RDW. A gradual increase in RDW with age has been widely reported [23]. Nevertheless, the results of this random study demonstrated that CTD-ILD was more likely to occur in older men than in younger men, which disproved our previous assumption that CTD is prevalent in women. Finally, no further analysis was performed in a subgroup of patients with CTD-ILD. Whether differences exist among subgroups remains to be further explored by data collection.

## 5. Conclusion

Patients with CTD-ILD have higher RDW levels compared with those with only CTD. RDW, a readily available, convenient, and cost-neutral test, is a promising tool for the screening and the prognostic stratification of ILD in patients with CTD.

## Figures and Tables

**Table 1:** Baseline characteristics of all patients with CTD-ILD.

Variable	CTD without ILD (*N* = 202)	CTD with ILD (*N* = 180)	*P*
Age, years (mean ± SD)	49.93 ± 13.76	52.91 ± 11.58	0.024
Sex, M/F	19/183	31/149	0.024
Hypertension	23	31	0.102
Diabetes mellitus	7	7	0.826
Heart disease	9	15	0.119
RDW (%)	14.17 ± 1.63	14.65 ± 2.08	0.012
WBC (×10^9^/l)	5.94 ± 2.94	6.22 ± 3.29	0.382
Alb (g/L)	35.24 ± 5.68	35.35 ± 5.39	0.847
LVEF (%)	64.78 ± 9.76	66.51 ± 4.80	0.049
HB (g/L)	108.28 ± 15.44	112.17 ± 17.38	0.022
Hospital confinement (days)	11.84 ± 5.40	12.60 ± 5.00	0.152
SOD (U/ml)	114.63 ± 21.05	116.65 ± 20.39	0.369
PASP (mmHg)	26.80 ± 5.91	30.92 ± 11.12	0.001
IgG (g/L)	14.30 ± 5.28	16.13 ± 5.44	0.002
C3 (g/L)	0.87 ± 0.30	0.94 ± 0.27	0.034
Hospitalisation expenses (RMB)	7271.39 ± 4955.46	9062.32 ± 6129.01	0.002
Number of readmissions in one year	1.21 ± 0.51	1.36 ± 0.90	0.038
Smoking status, ever/never	9/193	8/172	0.99

*N*: number; CTD: connective tissue diseases; ILD: interstitial lung disease; age is presented as median ± SD; RDW: red cell distribution width; WBC, white blood cell; ALB: albumin; LVEF: left ventricular ejection fraction; Hb: hemoglobin; SOD: superoxide dismutase; PASP: pulmonary artery systolic pressure; IgG: immunoglobulin G; C3: complement 3.

**Table 2:** Correlations between RDW and clinical and laboratory parameters in CTD-ILD.

Variable	RDW	*P*
HB (g/L)	*r* = −0.252	0.001
Plt (×10^9^/l)	*r* = −0.183	0.014
Alb (g/L)	*r* = −0.274	0.001
SOD (U/ml)	*r* = −0.274	<0.001
PASP (mmHg)	*r* = 0.349	<0.001
Hospital confinement (days)	*r* = 0.172	0.022
Hospitalisation expenses (RMB)	*r* = 0.158	0.037

Plt: platelet.

**Table 3:** Logistic regression analysis of CTD-ILD.

Variable	Univariate			Multivariate		
	OR	95% CI	*P*	OR	95% CI	*P*
WBC (×10^9^/l)	1.030	0.965–1.099	0.382			
RDW (%)	1.163	1.029–1.313	0.025	1.232	1.053–1.442	0.019
PDW (%)			0.167			
Hb (g/L)	1.015	1.002–1.027	0.023	1.017	1.000–1.034	0.036
Plt (×10^9^/l)	0.998	0.995–1.000	0.193			
ESR (mm/h)			0.593			
CRP (mg/L)			0.888			
Alb (g/L)			0.846			
ALT (U/L)			0.277			
AST (U/L)	1.004	1–1.009	0.065			
BUN (mmol/L)			0.585			
Cr (*μ*mol/L)			0.274			
RFn (IU/ml)			0.839			
IgA (g/L)	1.171	0.981–1.398	0.080			
IgG (g/L)	1.065	1.024–1.109	0.042	1.103	1.051–1.159	<0.001
IgM (g/L)			0.952			
IgE (IU/ml)			0.99			
C3 (g/L)	2.349	1.064–5.186	0.045	1.680	0.669–4.212	0.570
C4 (g/L)	8.986	0.975–82.826	0.073			
PASP (mmHg)	1.062	1.002–1.104	0.022			0.058
LVEF (%)	1.036	0.997–1.077	0.078			
Age, years	1.018	1.002–1.035	0.035	1.032	1.010–1.054	0.044
Sex (male)	2.004	1.008–3.690	0.036	2.104	0.915–4.837	0.180

PDW: platelet distribution width; ESR: erythrocyte sedimentation rate; CRP: C-reactive protein; ALT: alanine aminotransferase; AST: aspartate aminotransferase; BUN: blood urea nitrogen; Cr: creatinine; RF: rheumatoid factor; IgA: immunoglobulin A; IgM: immunoglobulin M; IgE: immunoglobulin E; C4: complement 4.

## Data Availability

The data sets used during the present study are available from the corresponding author upon reasonable request.
